# THE ROLE OF SOCIAL RELATIONSHIP PARTNERS FOR WELL-BEING AFTER SOCIAL INTERACTIONS IN DIFFERENT MODALITIES

**DOI:** 10.1093/geroni/igae098.2225

**Published:** 2024-12-31

**Authors:** Gizem Hueluer, Kaila Borgards, Birthe Macdonald

**Affiliations:** University of Bonn, Bonn, Nordrhein-Westfalen, Germany; University of Bonn, Bonn, Nordrhein-Westfalen, Germany; University of Zurich, Zurich, Zurich, Switzerland

## Abstract

Social relationships and social interactions are essential for psychological well-being. In the present study, we examine the role of social relationship partners (relationship closeness and social network size) for well-being after social interactions in different modalities (face-to-face, telephone, text-based digital). We use 7,861 observations from 113 participants (age: M = 72 years, SD = 5, range = 65 to 94; 40% women), who completed a short questionnaire about their social interactions over 21 days in an event-contingent experience sampling study. With regard to main effects, our findings showed that within persons, participants reported higher levels of well-being after social interactions with closer vs. less close social interaction partners. Social network size was unrelated to well-being after social interactions. Well-being was highest after face-to-face social interactions, followed by telephone and digital text-based social interactions. With regard to moderation effects, the effect of relationship closeness on well-being was not moderated by social interaction modality. The effect of social network size on well-being after social interactions was moderated by social interaction modality, indicating that the difference in well- being experienced after face-to-face and telephone vs. text-based digital social interactions was smaller for participants with smaller social networks. In summary, our findings show that social interactions with more close social partners are associated with higher well-being regardless of modality and that digital text-based communication may play a compensatory role for older adults with smaller social networks. We discuss implications of these findings for future research.

